# Modeling and forecasting the COVID‐19 pandemic time‐series data

**DOI:** 10.1111/ssqu.13008

**Published:** 2021-08-07

**Authors:** Jurgen A. Doornik, Jennifer L. Castle, David F. Hendry

**Affiliations:** ^1^ Nuffield College, Oxford, UK; ^2^ Magdalen College, Oxford, UK; ^3^ Climate Econometrics and Institute for New Economic Thinking at the Oxford Martin School, University of Oxford, Oxford, UK

**Keywords:** Covid‐19, epidemiology, nonstationarity, reproduction number, time‐series forecasting

## Abstract

**Objective:**

We analyze the number of recorded cases and deaths of COVID‐19 in many parts of the world, with the aim to understand the complexities of the data, and produce regular forecasts.

**Methods:**

The SARS‐CoV‐2 virus that causes COVID‐19 has affected societies in all corners of the globe but with vastly differing experiences across countries. Health‐care and economic systems vary significantly across countries, as do policy responses, including testing, intermittent lockdowns, quarantine, contact tracing, mask wearing, and social distancing. Despite these challenges, the reported data can be used in many ways to help inform policy. We describe how to decompose the reported time series of confirmed cases and deaths into a trend, seasonal, and irregular component using machine learning methods.

**Results:**

This decomposition enables statistical computation of measures of the mortality ratio and reproduction number for any country, and we conduct a counterfactual exercise assuming that the United States had a summer outcome in 2020 similar to that of the European Union. The decomposition is also used to produce forecasts of cases and deaths, and we undertake a forecast comparison which highlights the importance of seasonality in the data and the difficulties of forecasting too far into the future.

**Conclusion:**

Our adaptive data‐based methods and purely statistical forecasts provide a useful complement to the output from epidemiological models.

On March 11, 2020, the WHO declared that the SARS‐CoV‐2 virus causing COVID‐19 had become a pandemic. Since then, infections have overwhelmed many health systems, and sadly in some countries, also mortuaries. As accurate forecasts could help health and public authorities plan better, in mid‐March 2020, the authors started to produce short‐term forecasts for the number of recorded cases and deaths from COVID‐19, producing about four updates a week on www.doornik.com/COVID‐19. We now have experience in producing regular forecasts for over a year, so in this article we discuss the main measurement and methodological challenges faced when modeling and forecasting COVID‐19 data.

We outline a methodology that can handle the highly nonstationary nature of the pandemic time series. The COVID‐19 time‐series observations pose unique challenges compared to many other categories of time‐series data. There is an interplay between the health effects of the mutating virus and the societal and economic responses. This interaction between the epidemiological evolution of the corona virus with changing behavior and policy responses creates pandemic observations that are highly nonstationary with rapidly changing trends, sudden distributional shifts, outliers, measurement errors and revisions, and changing definitions of cases and resulting deaths, all of which vary from country to country. Furthermore, there are many issues with the quality and interpretability of the data. Such data characteristics require careful analysis in order to produce reliable short‐term forecasts, interpret mortality and reproduction rates, and undertake counterfactual analyses.

Our focus is on the number of confirmed cases and attributed deaths as the variables of interest as these are readily available for countries and smaller geographical areas. These are also the main variables of interest in the media and for policy decisions. We apply a decomposition methodology that uses a machine learning algorithm to extract a highly flexible trend which can handle both smooth changes and abrupt breaks, along with a “seasonal” (here systematic weekly variation) and an irregular component. Each of these components is forecast separately then combined but dampening their trends. Using such a flexible decomposition methodology enables careful analysis of nonstandard time‐series data and gives key insights into how to handle such data if faced with future crises and pandemics.

We commence by describing the data, which highlights the many sources of nonstationarity that must be dealt with, including the rapidly evolving and sometimes explosive trends, the strong seasonality (initially unanticipated), substantial measurement errors and major data revisions including accounting mistakes and changing definitions, as well as structural breaks from interventions like lockdowns. A discussion of the different experiences of the European Union, Latin American Countries (LAC), and United States serves to emphasize that political, economic, and institutional forces are important factors in pandemic outcomes, and hence any methodology must be sufficiently flexible to handle such differences. We then outline the methodology of our approach, describing the statistical decomposition, before discussing how to compute a simple measure of the mortality ratio from the extracted trend, enabling cross country or regional comparisons. This is followed by a section that outlines a measure of the reproduction rate, which describes how rapidly the disease spreads, using the statistical decomposition. We then undertake a counterfactual study that hypothesizes what the U.S. death tally would have been if it had experienced the same rates over the summer of 2020 as the European Union. The last three sections prior to the conclusions relate to forecast challenges and evaluation. After considering the challenges of forecasting the number of cases and deaths, we compare our statistical forecasts to those based on epidemiological models, then consider how far ahead it is feasible to forecast at all accurately.

## DATA

Our initial forecasts of the number of cases and deaths covered 28 countries, but as the pandemic spread across the world, this extended to about 50 countries, 50 U.S. states, and more than 300 local authorities in England. We write $I_{t}$ for the cumulative number of cases and $i_{t}$ for the daily number of newly infected individuals, in so far as they are observed from a positive test:

$$i_{t}=I_{t}-I_{t-1}\equiv \Delta I_{t}.$$

The cumulative number of deaths is $D_{t}$, with the daily count $d_{t}=\Delta D_{t}$. All our results are obtained from data supplied by Johns Hopkins University Center for Systems Science and Engineering (JH/CSSE, see Dong, Du, and Gardner, 2020).

The JH/CSSE data dashboard reports $I_{t}$ and $D_{t}$, while the European Centre for Disease Prevention and Control reports $i_{t},d_{t}$ as well as the “cumulative number for 14 days of COVID‐19 cases per 100,000,” which is $I_{t}-I_{t-14}$ divided by population/100,000, where population is kept fixed.

**FIGURE 1:**
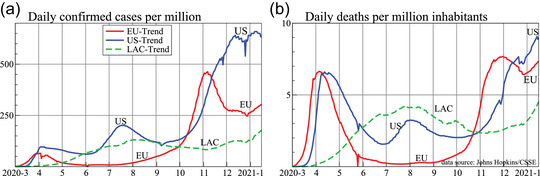
Daily confirmed cases (a, left) and deaths (b, right), both per million inhabitants *Note*: Plotted is the estimated underlying trend with seasonality removed.

Figure 1 shows the smoothed trends from the reported data using our trend decomposition methods in the European Union, LAC, and United States, in each case reported per million inhabitants.^1^ These three large regions account for roughly 20 percent of the world's population. The estimated trend in daily confirmed cases is in the left panel (labeled E.U.‐Trend for the European Union, etc.), and deaths on the right. In each case, we have removed effects that are specific to the day of the week (seasonality) and short‐term random fluctuations (described below).

The experiences of the three regions differ markedly, with varying growth rates and nonsynchronous peaks, showing that political, economic, and institutional forces are important factors in pandemic outcomes. Furthermore, the response has had a major economic impact. In January, the IMF expected global growth to be about 3.3 percent in 2020 (World Economic Outlook, WEO, Update, January 2020). By April, this was revised down to a 3 percent fall, and the October WEO forecast is lower still with a drop of 4.4 percent. Such economic implications feed back into public health decisions, leading to endogeneity between the economic and health systems, which give rise to further nonstationarities from interventions.

The pandemic data have been subject to substantial revisions, occasional errors (such as the naive spreadsheet error in the United Kingdom, causing 16,000 cases to be lost for a week), and accusations of political attempts to keep the numbers down. In the case of Brazil there was even a short‐lived attempt to prevent their publication (see The Lancet Editorial, 2020). We now provide examples of data revisions that any methodology must handle when modeling the data.

**FIGURE 2:**
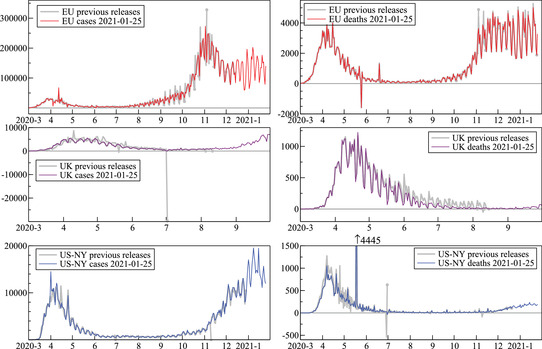
Data revisions for the European Union, United Kingdom, and New York State *Note*: Daily confirmed cases (left) and deaths (right).

We previously recorded large revisions in the data for March and April 2020 (Doornik, Castle, and Hendry, 2020a). Unfortunately, this has remained a prominent feature of subsequent data. Figure 2 plots in the top row the daily confirmed cases and deaths for the European Union. The thin line shows the most recent data release (from January 25, 2021). Previous releases that are different are shown in light gray, ending in a solid circle, but only for releases after June 1, 2020. Thus, the April 2020 data still changed after June, and the September and October data were also subject to large revisions. There were some small negative case counts, and one large negative death count (Spain, partially offset a month later).

The United Kingdom had a large downward revision in the number of cases at the start of June 2020, and prominent revisions to deaths. However, from September there are no further changes. U.S. data were also subject to revisions, as shown here for the state of New York. A massive spike of 4445 deaths appeared in May 2020, as well as a data release with a large negative death count followed by a large positive one at the end of June.

The data also have strong weekly patterns (seasonality), but not always (as for U.K. cases), and these also change over time and from revisions. The outliers, mistakes, and corrections in the data complicate modeling and forecasting.

## METHODOLOGY

Our approach to forecasting is purely statistical: the observed cumulative time series are decomposed into an underlying flexible trend, seasonal component, and a remainder term. The trend and seasonal are estimated by taking relatively short moving windows of the data and saturating these by segments of linear trends (trend indicator saturation, TIS). Selection from these linear trends is made with an econometric machine learning algorithm (Doornik, 2009), and the selected sparse subset estimates are then averaged to give the overall flexible trend and seasonal. Next, the trend and remainder terms are forecast separately using the “Cardt” method (Castle, Doornik, and Hendry, 2021) and recombined in a final forecast (details are in Doornik, Castle, and Hendry, 2020a with an updated assessment in (Doornik, Castle, and Hendry, 2020b).

Let $y_{t}$ denote cumulative cases $I_{t}$ or cumulative deaths $D_{t}$. Our forecasting models are for the logarithm of $y_{t}$. The first step is to decompose $y_{t}$ into trend ${\hat{y}}_{t}$, seasonal ${\hat{\gamma }}_{t}$, and remainder ${\hat{u}}_{t}$. Negative numbers are not an issue with the cumulative counts. But counts are zero at the start of the pandemic, which would prevent the use of logarithms. To handle this, counts are increased by one. The resulting decomposition for observations t=1,…,T is

$$\log\left(y_{t}+1\right)=\log\hat{\mu }+\log{\hat{\gamma }}_{t}+{\hat{\varepsilon }}_{t},$$

which is transformed back, using ${\hat{u}}_{t}=\exp\left(\;{\hat{\varepsilon }}_{t}\right)$ and setting $\hat{\mu }={\hat{y}}_{t}+1$:

(1) $$y_{t}=\left({\hat{y}}_{t}+1\right)\;{\hat{\gamma }}_{t}\;{\hat{u}}_{t}-1=\;{\hat{y}}_{t}\;{\hat{\gamma }}_{t}\;{\hat{u}}_{t}+\left[{\hat{\gamma }}_{t}\;{\hat{u}}_{t}-1\right]\approx {\hat{y}}_{t}\;{\hat{\gamma }}_{t}\;{\hat{u}}_{t}.$$

Both ${\hat{\gamma }}_{t}$ and ${\hat{u}}_{t}$ have an expectation of one and are uncorrelated. Using logarithms results in a multiplicative decomposition.

Saturation with the linear trends is in terms of the first differences of $\log(y_{t}+1)$, with the resulting trend and seasonal components reintegrated to the levels. We called this the DL‐TIS specification of the model. Then Cardt forecasts are made of the trend and irregular, while the seasonal forecasts are kept fixed at the estimated values for the previous week.

The estimated daily trends (as in Figure 1) could be derived as the daily change of the cumulative trends used to forecasts. However, those trends are more noisy than desired, so instead we work directly with the daily counts $i_{t}$ and $d_{t}$. Our preference is to apply the methodology to $\log i_{t}$ and $\log d_{t}$. The prevalence of zeros can be high. For example, starting in July, Spain only reports counts during the week, and had spells with no deaths in summer 2020. This is handled by the small adjustment of adding unity, and so the model is denoted L‐TIS. The negative numbers are more serious, requiring either removal, or an additive decomposition:

(2) $$y_{t}={\hat{y}}_{t}+{\hat{\gamma }}_{t}+{\hat{u}}_{t},$$

where in (2) ${\hat{\gamma }}_{t}$ and ${\hat{u}}_{t}$ will both be zero on average.

**FIGURE 3:**
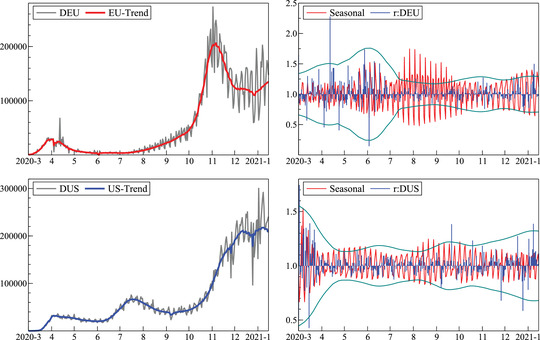
Daily confirmed cases: Estimated trend with observed data (left), residuals (right: r:DEU, r:DUS) and seasonals (right) *Note*: European Union (top) and United States (bottom).

Figure 3 shows ${\hat{i}}_{t}$ and $i_{t}$ on the left, and $\hat{\gamma }{\;}_{t}^{i}$ and $\hat{u}{\;}_{t}^{i}$ on the right (denoted r:DEU and r:DUS). The top row is for the European Union and the bottom for the United States, for the data release of January 25, 2021 using the multiplicative model (small negative case counts for the United States were set to zero). The remainder $\hat{u}{\;}_{t}^{i}$ is plotted with time‐varying 95percent confidence bands. Seasonality for the United States is relatively constant from May onward. The European Union has no seasonality in March and April, then regular seasonality for two months, and a more complex and pronounced pattern from July onward, changing again in the autumn. The pandemic starts later in the United States, which is reflected in the larger initial errors.

**FIGURE 4:**
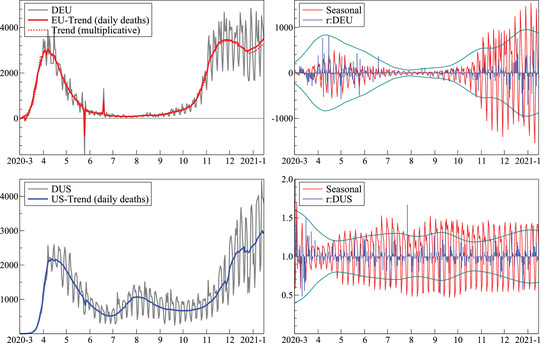
Daily deaths: Estimated trend with observed data (left), residuals and seasonals (right) *Note*: European Union (top) and United States (bottom).

Figure 4 shows the equivalent results for daily deaths. The E.U. decomposition uses the additive decomposition defined in (2) in order to handle negative numbers. The implication of this decomposition is that the residuals and seasonal are on a very different scale, and more heteroskedastic as the log transformation reduces heteroskedasticity, particularly when the error variance is proportional to the level of the variable. The first plot also includes the trend from the multiplicative model (1), obtained by setting the large negative count to 0. This difference is hard to see, except that the multiplicative trend is lower at the end. The second difference is that the upward spike is located in the residuals, and the downward spike is lower (by construction). So outliers can end up in the residual term, or remain in the trend, where they can be removed later. The difference from other smoothing procedures is that the outliers remain identifiable: they are localized rather than smoothed over.

## MORTALITY

In this section, we use our statistical decomposition to derive a measure of the case mortality ratio (CMR), enabling cross‐country and region comparisons. Such a measure could be used for forecasting or data reconstruction due to measurement errors, as well as being informative about the varying experience of the severity of the virus both across countries and over time. It may also aide in measuring the impact of vaccination.

The case fatality rate (CFR) is described as “The proportion of cases of a specified condition that are fatal within a specified time” (in Porta, 2013). This is a number between 0 and 1. Computation of the CFR requires determination of a case, as well as a time frame until resolution (recovery or death). Incorporating all cases requires knowledge of the infection rate in the population, not just deaths from confirmed cases. There is no reason for the fatality rate to be constant: a cure will bring it down to 0; it also tends to be age specific and region specific. Addressing the first issue, WHO (2020) defines cases as confirmed cases. In contrast, the infection fatality rate (IFR) is then relative to the total number of infections. Sweden (2020) estimate the IFR in Stockholm for cases starting late March 2020 to be about 0.6 percent (0.1 percent up to 69‐year‐olds, 4.3 percent from 70‐year‐olds). WHO (2020) quotes estimates in the range 0.5–1 percent. Brazeau, Verity, and Jenks (2020) also find strong dependence of IFR on age, and estimate an average IFR of about 1 percent in Brazil, England, Spain, and Sweden.

A more simplistic version computes a CMR as

(3) $${\hat{m}}_{t}=\frac{{\hat{d}}_{t}}{{\hat{i}}_{t-14}}.$$

**FIGURE 5:**
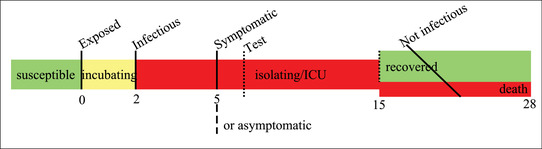
Approximate average time line of a SARS‐CoV‐2 infection from day 0 to day 28

This assumes an average delay of 14 days between testing and deaths; similar to 13 days (in Wilson et al., 2020) and 14 days (in Baud et al., 2020). The time line of a typical case, based on the summary information (in Siorda, 2020) is depicted in Figure 5. Also assumed is that all confirmed cases are resolved in two weeks. In addition, the denominator ignores cases that are not tested. The assumptions on timings, including cases being resolved in two weeks, are not essential to the computation of CMR. Alternative assumptions could be plugged into (3), but meaningful comparisons across countries or regions would require consistent assumptions for each comparator.

**FIGURE 6:**
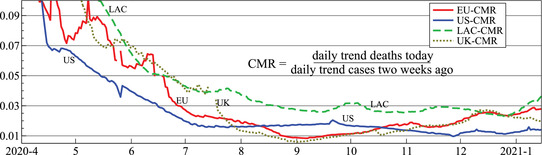
Ratio of deaths today to confirmed cases two weeks before, based on the estimated smoothed underlying trends

Figure 6 plots the CMR: the ratios of the underlying trend of daily deaths (see Figure 1b) to cases two weeks earlier (see Figure 1a) for the European Union, LAC, United States, and United Kingdom. This shows some similarities between the pandemic evolution in different parts of the world. There is a steady decline initially, after which the CMR more or less settles. The United States shows a steady and more or less constant decline in ${\hat{m}}_{t}$ up to early July, after which the ratio stabilizes. The European Union is more erratic, but with a similar overall pattern. The United Kingdom and European Union are close from September onward. Both are edging up, which could be related to intensive care pressures or variants with a higher mortality (or relaxation of testing). Similar trends apply to LAC, although the ratio is higher from July onward.

As noted before, this time‐varying mortality ratio is not similar to the fatality rate of SARS‐CoV‐2: the number of cases is just the number of positive tests, and not the number infected in the population. Moreover, while the number of deaths is probably a fairly accurate representation, the definition has changed in some countries, and has uncertain timing. The United Kingdom counts a COVID‐19 death as someone who dies with a positive test in the previous 28 days, but initially counted anybody with a positive test, regardless of cause of death or time from test. The initial decline of the ratio could be due to the gradual expansion of testing capacity, possibly aided by improved medical outcomes. The composition of the infected population may also have changed: when students returned to universities after vacations, the number of cases rose sharply, but students have a much lower IFR (Baud et al., 2020, estimates about 0.03 percent).

**FIGURE 7:**
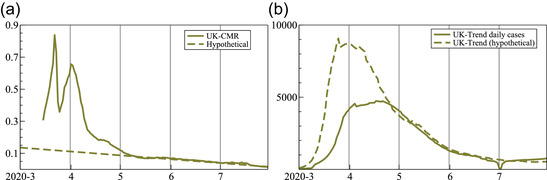
Actual and hypothetical CMR for the United Kingdom (a, left), and actual and implied daily number of cases (b, right)

The relatively slowly changing CMR, combined with the assumption that they are fairly accurately measured could form an alternative basis for forecasting deaths from cases. Or it could be used to reconstruct cases over a period when they were mismeasured (such as the period of the spreadsheet error in the United Kingdom). Figure 7a shows CMR of the United Kingdom. Then, if instead the CMR had followed the dashed hypothetical line, the corresponding number of daily cases would have been as shown in the dashed line on the right. This contrasts with the solid line that reports the actual trend, suggesting a peak about two weeks earlier and twice as high. Although this attributes most of the effect to a lack of testing, it is probably a more realistic representation of that period in the United Kingdom.

## THE REPRODUCTION NUMBER

The reproduction number indicates how many susceptible individuals are infected through contact with an infectious person. It summarizes the rate of growth of an epidemic: if the number is below 1, the incidence in the population is falling, while above 1 it is growing: if it is 2, then an infectious person infects two others, so the number of cases doubles. The reproduction number is an average for a population, and can hide substantial geographical and age‐related heterogeneity. It is not a quantity that is directly observed, but usually obtained as a by‐product from a model. In this section, we demonstrate how our statistical decomposition can be used to compute a measure of the reproduction rate.

The Robert Koch Institute in Germany reports the instantaneous reproduction number, $R_{t}$, derived from the number of confirmed cases, that is, positive SARS‐CoV‐2 tests. Their approach (Koch‐Institute, 2020) (building on Cori et al., 2013) computes $R_{t}$ using moving averages:

$$R_{t}=\frac{i_{t}+i_{t-1}+i_{t-2}+i_{t-3}}{i_{t-4}+i_{t-5}+i_{t-6}+i_{t-7}}=\frac{I_{t}-I_{t-4}}{I_{t-4}-I_{t-8}}=\frac{\Delta_{4}I_{t}}{\Delta_{4}I_{t-4}},$$

assuming that individuals remain infectious for four days, that is, the time from infection to test result (and isolation) in Figure 5. Like CMR before, this measure is derived from directly observed quantities, and simpler than the estimates obtained from mathematical models.

With seasonality following a pattern within the week, this definition will remain highly seasonal. To remove the seasonality, Koch‐Institute (2020) suggests using

$${\bar{R}}_{t}=\frac{\Delta_{7}I_{t}}{\Delta_{7}I_{t-4}},$$

which has the drawback of adding an additional delay of three days to the instantaneous reproduction number. To avoid this, we use ${\hat{i}}_{t}$ from our model for $i_{t}$ to define $R_{t}$ as

$${\hat{R}}_{t}=\frac{{\hat{i}}_{t}+{\hat{i}}_{t-1}+{\hat{i}}_{t-2}+{\hat{i}}_{t-3}}{{\hat{i}}_{t-4}+{\hat{i}}_{t-5}+{\hat{i}}_{t-6}+{\hat{i}}_{t-7}},$$

which also has seasonality removed. In common with Harvey and Kattuman (2020), we can use time‐series forecasts of $i_{t}$ to get more up‐to‐date estimates of the current instantaneous reproduction number. Uncertainty estimates can be derived from the time‐series decomposition; the values that are based on forecasts will be less certain.

**FIGURE 8:**
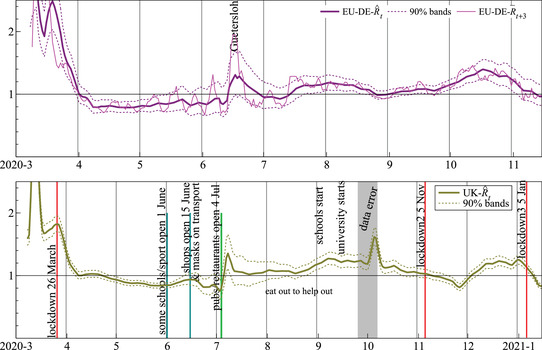
Estimates of the reproduction number for Germany (EU‐DE, top) and the United Kingdom (UK, bottom)

The top panel of Figure 8 has the estimated instantaneous reproduction number for Germany as the solid line together with approximately 90 percent uncertainty bands. Also shown as a thin dotted line is ${\bar{R}}_{t}$ with a three‐period lead to center it on the central date. The two estimates are similar, but those based on the trend decomposition are less noisy. Both show the mid‐June increase from the meat‐plant infections in Gütersloh. ${\hat{R}}_{t}$ is more responsive than that seen in many other models.

The bottom part of Figure 8 shows the evolution of ${\hat{R}}_{t}$ for the United Kingdom, together with dates of some prominent mitigation measures and events for England.^2^ Superficially at least, there seems to be some relation between these events and a change in the reproduction number. The area around the start of October is marked as a data error. Using an obsolete Excel spreadsheet file format, almost 16,000 cases went unreported between 25 September 25 and October 2, added back in on October 3 and 4. Through the formula for ${\hat{R}}_{t}$, a single‐day error first spends four days in the numerator, then another four in the denominator. This leads to the fall, then steep rise in the reproduction number. The timing was unfortunate because it may have hidden a rise in cases, just when the second wave was getting established.

## A COUNTERFACTUAL STUDY

We saw in Figure 1 that the United States had a second wave in the summer, but the European Union did not. As a counterfactual study, we hypothesize what the U.S. death tally would have been if it had the summer rates of the European Union. We do not claim to elicit causal inference from this counterfactual example. Such an approach would require a formal structural causal model, (see, e.g., Pearl, 2009), which is outside the scope of our statistical decomposition analysis, but it is intended to provide a summary measure of the number of U.S. deaths had the United States followed the same pattern as the European Union. To align pandemic evolution up to the middle of April, we multiply E.U. cases by 1.5 and add a delay of eight days. This keeps the actual and counterfactual the same up to the start of May. Next, we assume that the U.S. ratio ${\hat{m}}_{t}$ was kept at its measured values. The counterfactual U.S. death count then is

$${\tilde{d}}_{t}^{\;\text{US}}=\frac{330}{445}1.5\;{\hat{i}}_{t-22}^{\;\text{EU}}\;{\hat{m}}_{t}^{\;\text{US}},$$

where the lag length of 22 consists of the assumed eight‐day delay plus two weeks in the mortality ratio. The ratio of 330/445 is the population adjustment.

**FIGURE 9:**
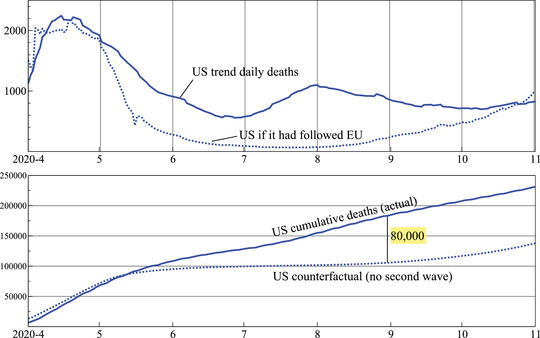
Counterfactual study of deaths in the United States: What if the United States avoided its second wave and instead had a summer like the European Union

Figure 9 shows the outcome of the counterfactual study using the data release from November 5, 2020. Based on the assumptions that we made, if the United States had avoided the second wave in the summer of 2020, as the European Union did, it would have avoided about 80,000 deaths. Reducing the adjustment factor of 1.5 would increase this number.

It is tempting to attribute this excess mortality to political differences. However, there are other factors in play. The United States has a younger population with a median age of 38.1 years versus 42.9 for the European Union (source: Wikipedia), although COVID‐19 mortality rises with age. On the other hand, the United States has higher obesity than all European countries, and this is correlated with the three main comorbidity factors (hypertension, diabetes mellitus, and cardiovascular disease: see Siorda, 2020). The OECD reports that in 2015, 23.6 percent of Germans were overweight compared to 38.2 percent of Americans, which could be used as a rationale for the factor 1.5 in the expression above.

## FORECASTING CHALLENGES

In this section, we present our forecasts using the methodology described above. We focus on the six largest LAC (excluding Venezuela), denoted LAC6.^3^ The data for these six countries exhibit characteristics that emphasize challenges for both modeling and forecasting, including large jumps, erratic data collection, and changing seasonality. The countries differ widely in their testing strategies, health institutions, mitigation strategies, economic measures, and political approach, all of which introduce nonstationarities in the data generating process that are in addition to the stylized epidemic curve of exponential growth that flattens. Despite these challenges, it is useful to have forecasts to aid in planning, and several institutions supply and publish them.

Furthermore, these countries are of direct interest given the economic impact of COVID‐19. In October, the IMF wrote that “Regional differences remain stark, with many countries in Latin America severely affected by the pandemic facing very deep downturns” (WEO October 2020). The GDP forecast for Brazil in 2020 was −5.8 percent, which would leave GDP more or less where it was 10 years ago. The situation for Argentina is even starker, with a predicted decline of −11.8 percent after a decade of no growth. Hence, there is significant interest in forecasts for LAC countries.

**FIGURE 10:**
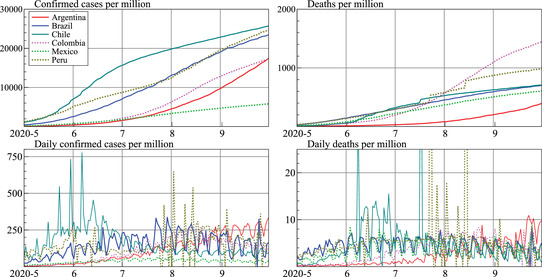
Confirmed cases and deaths per million inhabitants in LAC6 (top row); Daily cases and deaths (bottom row)

Figure 10 shows cumulative counts and deaths per million for each country in LAC6 along the top, and their daily counts and deaths along the bottom for the period from May to September 2020. The smoother cumulative counts hide large differences between countries in weekly patterns and growth rates. Peru, in particular, has several large upward jumps in deaths (extending beyond the visible part of the bottom‐right plot). As a comparison with our forecasts, we include the forecasts provided by the Los Alamos National Laboratory (LANL, see covid‐19.bsvgateway.org) in our next two graphs.^4^

**FIGURE 11:**
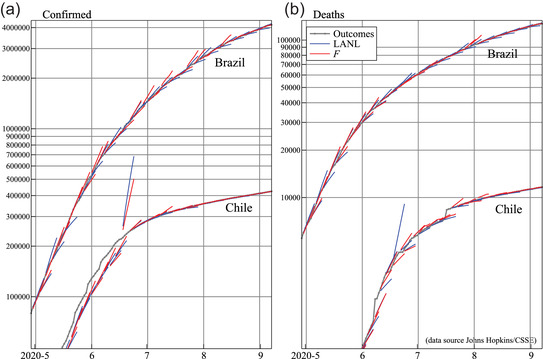
Forecast paths of cases (left) and deaths (right) for May to early September for Brazil and Chile *Note*: Our forecasts denoted F, together with LANL forecasts.

Figure 11 shows the paths of our forecasts (F) and LANL on a logarithmic scale for Chile and Brazil. It also shows the outcomes as reported in early November, in the thicker line with dots. Toward the end, as the pandemic growth diminishes, all forecasts get better, although we can discern more blue lines appearing below the outcomes.

For Chile, we see that the forecasts are below the outcomes at first: the reason is that the data were revised on a later date. Initially that revision was not implemented backward, explaining the strong upward forecast path of confirmed cases in the middle of June: the data suggested a sudden explosion in the number of confirmed cases. This jump did not appear in observed deaths, but is present in the LANL forecasts of deaths (Figure 11b), showing that the model links both.

**FIGURE 12:**
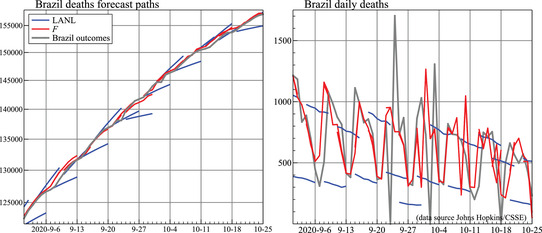
Forecast paths of deaths (left) and daily deaths (right) for September and October for Brazil *Note*: LANL and our forecasts F.

Figure 12 zooms in on the Brazil forecasts for September and October. This shows that incorporating seasonality in the model can make a huge difference to forecast accuracy. All LANL forecasts have the first forecast either on Monday or on Thursday. The former start from a weekly low, so consistently underforecast. The midweek forecasts start at a high, so overforecast. The forecasts F track the outcomes more closely because the model allows for the weekly pattern: plotting the differenced forecasts and outcomes highlights this in the right‐hand panel. Our initial models did not allow for seasonality: it was not visible at the start, and we did not expect it. We incorporated weekly seasonality in our models from early July, and LANL updated their model in November.

## FORECAST EVALUATION

Our forecasts are statistical extrapolations intended to inform policy decisions, which in turn hopefully lead our forecasts to be wrong, as we are not modeling and forecasting medical or nonpharmaceutical interventions. If our forecasts are wrong, say from a successful vaccination program, it could still demonstrate their usefulness as counterfactual scenarios. Nonetheless, we produce very regular forecasts that are adaptive to changes as they occur, so it is also of value to assess the accuracy of our forecasts against the outcomes.

Interval forecasts convey the uncertainty of the forecasts and provide essential information. Think of these as “health warnings” as seen on pharmaceutical packages such as “taking paracetamol may leave you feeling drowsy.” An equivalent health warning for any published forecast would be to clearly state the width of the interval forecasts (at, say 95 or 90 percent), and the conditioning information used in their preparation. Not only that, but the uncertainty is itself uncertain, again this needs to be conveyed. It can also be evaluated afterward.

${\hat{D}}_{t|s}$ are the forecasts for date t of cumulative deaths, made on date *s* < *t*. Then $D_{t}-{\hat{D}}_{t|s}$ is the forecast error $e_{t}$, and $100e_{t}/D_{t}$ the forecast error as a percentage of the outcome. We use two relative summary measures of forecast accuracy: the mean of the absolute values of the percentage errors (MAPE, also see Doornik, Castle, and Hendry, 2020a), and the median of the absolute percentage errors (MedAPE). The MedAPE uses a robust measure of the mean, and so is less sensitive to a rare but large error. The third measure is the median of absolute errors (MedAE) (absolute errors are preferred by Bracher et al., 2021). Other measures are possible, and all may give different rankings: none provide measures of the statistical significance of forecast difference. Previous results showed good forecast performance of our methods in comparison with three others (for March–April 2020 in Doornik, Castle, and Hendry, 2020a), and (for May–September in Doornik, Castle, and Hendry, 2020b).

The U.S. CDC provides forecasts for U.S. states from a wide range of forecasters, up to several weeks ahead. Our forecasts do not qualify for inclusion because we only go up to one week ahead. The Reich Lab at the University of Massachusetts Amherst constructs ensemble forecasts out of the included forecasts (Ray, Wattanachit, and Niemi, 2020). Forecasts of deaths are available from April 2020, and cases from August. We can compare the forecasts provided by the CDC to ours, but only for one week ahead.

Previously, we only included forecasts for the same date, horizon, and locality in the accuracy measures for optimal comparability of two sets of forecasts. Because there are many models in the CDC set, we relax that requirement. We adopt some rules to increase comparability of results:

- Only include forecasts when there are at least 100 deaths or 1000 cases. This avoids forecasting when the counts are low and the percentage errors could be massive.
- Included are the 50 states and District of Columbia, but the accuracy measures are aggregated over these using population weights. So, small states (Rhode Island data are particularly lumpy) do not dominate the results.
- Forecasts are compared to the earliest available outcome for the target date from JH/CSSE daily reports for U.S. states.^5^ We exclude models that have a relatively small number of forecasts: the percentage error is expected to decrease over time as the totals go up and information accrues. This limit is less strict for cases, where there are fewer consistent forecasters.

Table 1 gives a summary of the results. The initial columns consist of the rank (based on MedAPE), the count (number of one‐week‐ahead forecasts included), MedAPE, MedAE, and MAPE. The last two columns measure the coverage: the percentage of outcomes that are outside the interval forecasts, either below or above. The table includes our main forecasts “*F*,” as well as our “Avg” forecasts. Avg is constructed out of forecasting with two model specifications commencing from the last four data points, so conditioning only on actual outturns four days previously, three days previously, etc. These are then adjusted to match the last known observation and averaged. Also shown are the CDC ensemble model, and the top and bottom ranking model, based on MedAPE. “Average of models” gives the average values over all the models included, and is given the rank of where its MedAPE would slot in. The average coverage excludes *F* and Avg.

**TABLE 1:** Evaluation of one‐week ahead forecasts for cumulative deaths and incremental cases, aggregated over U.S. states

	Rank	Count	MedAPE	MedAE	MAPE	%Below	%Above
Deaths: 16 models of 25 (≥150 forecasts, May 16, 2020 to July 4, 2020)							
Top model	1	329	1.49	32	1.88	3.3	11.6
Ensemble model	3	329	1.72	39	2.16	2.7	2.1
*F*	4	269	1.87	60	2.77	9.3${}^{a}$	5.2${}^{a}$
Avg	6	269	1.91	67	2.74	7.8${}^{a}$	6.3${}^{a}$
Average of models	12	283	2.92	77	3.91	13.5	13.8
Bottom model	16	248	5.46	150	7.60	0.0	19.2
Deaths: 30 models of 48 (≥700 forecasts, August 8, 2020 to January 2, 2020)							
Ensemble model	1	1050	0.85	61	1.10	1.9	8.8
Avg	6	864	0.89	57	1.32	4.3${}^{a}$	23.6${}^{a}$
*F*	11	864	0.97	69	1.51	7.3${}^{a}$	22.9${}^{a}$
Average of models	21	942	1.16	85	1.55	10.6	22.3
Bottom model	30	786	2.19	170	2.98	19.2	54.7
Cases: 29 models of 41 (≥300 forecasts, August 8, 2020 to January 2, 2021)							
Top model	1	549	0.66	1750	0.95	56.2	5.8
Ensemble model	8	1049	0.94	3190	1.27	2.5	14.9
*F*	19	992	1.34	4330	1.86	7.6${}^{a}$	26.7${}^{a}$
Average of models	22	773	1.37	5310	1.81	18.3	22.3
Avg	22	992	1.37	4440	1.89	5.2${}^{a}$	27.8${}^{a}$
Bottom model	29	350	3.15	13,060	3.54	27.7	27.7

${}^{a}$ *F* and Avg use 80 percent intervals and are excluded from the average of models. The rest use 95 percent.

The top two panels of Table 1 are for deaths, based on reported forecasts of cumulative deaths one week ahead, covering the earlier and later period. As expected, forecast‐averaging helps, placing the ensemble model close to or at the top. Our forecasts also show excellent performance. As expected, the percentage measures are lower for the later period, effectively putting more value on good forecasting in the early stages of the pandemic. For May–June, the rankings are largely unaffected by using MedAE. But for August–December, Avg has the lowest value of the 30 included models (and second lowest out of 48).

For Avg and *F*, the intervals are given at 80 percent, so we hope to see 10 percent below and 10 percent above. All other models have a 95 percent interval, so target 2.5 percent outside the intervals on either side. For May–June, *F*, Avg, and especially the ensemble, perform well in this respect. Coverage in the later period is not so good, and most models struggle to provide reliable intervals.

The bottom panel of Table 1 is for cases, but by construction not directly comparable to deaths: the CDC files for cases only include weekly increments, and cumulative cases are constructed by adding the observed base. So, these are not the actual forecasts of the levels (as we can confirm from deaths when both are available). One plausible explanation is that many models take time to run, so are not based on the very latest information. In that case, the results for cases are more about forecasting potential, and those for deaths about the actual forecasting process. Our methods are fast, and our published forecasts are always based on results downloaded that same day, giving a possible process advantage. Now our forecasts are average performers. The bottom 10 percent quantile is fairly well captured, but the top quantile is too low. The coverage of most models is unsatisfactory.

Table 2 reports the summary measures for LAC6, based on a like‐for‐like comparison. Here a straightforward average is used over the nine months from May to December 2020. At horizons up to 3 there is not much difference, but beyond that F has a small advantage. Both forecasters struggle again to get the forecast intervals correct: an ideal outcome would show 10 percent outside at each horizon.

**TABLE 2:** One, three, and seven days ahead forecast evaluation May–December 2020; LAC6 (Brazil, Mexico, Colombia, Argentina, Peru, Chile)

	MedAPE		MAPE		%Below		%Above	
h	LANL	*F*	LANL	*F*	LANL	*F*	LANL	*F*
	Deaths							
1	0.19	0.25	0.66	0.74	19.7	4.2	23.4	4.8
4	0.64	0.48	2.05	1.82	10.7	3.7	25.1	9.0
7	0.99	0.67	3.10	2.66	6.5	5.4	23.7	11.3
	Cases							
1	0.15	0.29	0.49	0.67	14.1	5.6	16.3	2.5
4	0.73	0.69	1.88	1.79	7.6	9.6	22.0	5.9
7	1.20	1.01	3.14	3.06	6.8	12.1	20.8	7.0

*Note*: Intervals based on 0.1 and 0.9 quantiles.

## HOW FAR AHEAD CAN WE FORECAST?

We produce seven‐day ahead forecasts, shorter than most (10 days in Petropoulos and Makridakis, 2020). Others have produced forecasts at much longer horizons. For example, the Institute for Health Metrics and Evaluation (IHME: www.healthdata.org) produce forecasts for more than three months ahead, including scenario forecasts depending on universal mask usage, rapid vaccine rollout, and mandate relaxation. As well as producing the short‐term forecasts, LANL produces six‐week‐ahead forecasts with uncertainty bands. Some epidemiological models produce even longer term forecasts spanning an entire pandemic cycle.

**FIGURE 13:**
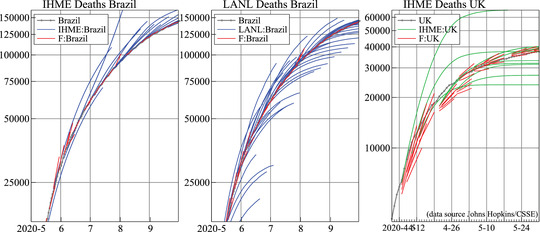
Long‐run forecast paths for deaths in Brazil IHME (left) and LANL (middle), U.K. deaths IHME (right)

Given the range of forecast horizons considered, a natural question is: how far ahead can we usefully forecast? There is a clear tradeoff; policymakers would welcome as long‐term forecasts as possible, but the further ahead the forecast, the larger the degree of uncertainty associated with the forecasts. If uncertainty bands were accurate, then forecast users could interpret the long‐range forecasts appropriately. But many long‐term forecasts are much less reliable than their reported intervals suggest. Figure 13 records the long‐run forecast paths for deaths from IHME and LANL for Brazil (along with our seven‐day ahead forecasts in the short lines), as well as the United Kingdom. The long‐run forecasts show huge deviations from the outturns, which are not reflected in long‐run forecast intervals. This suggests that we cannot forecast as far ahead as we think we can, or would like to. We may need to lower our belief in our models and be more circumspect. This is because the future will reveal many unanticipated events—the pandemic itself is one such, as are all the very different public, private, and medical responses to it. Few forecast uncertainty measures include an allowance for unpredictable changes, often a key source of the nonstationarity in pandemic data.

## CONCLUSION

Public‐health decision making in the pandemic relies on the reported data on cases and deaths, and so interpreting and forecasting the data in useful and accurate ways is essential. Although there is interesting information in the reported data, the data itself is messy, and robust methods are needed to interpret it. We propose a method of decomposition that extracts a flexible unobserved trend in the data that can be used to compute statistical measures of a simplistic mortality ratio and the reproduction number, as well as undertake *ex post* counterfactual analysis. Underpinning the analysis is the nonstationarity of the data, both with exponential trends and sudden shifts due to, e.g., mandating face coverings or lockdowns, combined with a nonstationary reporting methodology, with stochastic trends, such as the ramping up of testing, and distributional shifts, such as the sudden inclusion of care home cases. There is a compounding effect as the nonstationarity of the underlying data interacts with the nonstationarity of the reporting process. This requires methods that can handle stochastic trends and breaks jointly, which our proposed decomposition method does.

Despite the simplicity of our statistical approach, we can produce useful estimates of some key metrics that the epidemiological models focus on, including the reproduction number. Although our estimates are constrained by the quality of the reported data, they have the advantage of being data based rather than theory driven using unverifiable assumptions, as many of the epidemiological model estimates are. Furthermore, they are easy to compute for any country or region, allowing for cross‐country comparisons. While sensitive to data errors, this aspect can be revealing *ex post*.

Our aim was to provide short‐term forecasts of the numbers of confirmed cases and of deaths attributed to COVID‐19 as a guide to planning for the coming few days, so that the next reported numbers would not be a surprise. In doing so, we use robust methods that can handle the messy data available. We cannot avoid forecasting errors, but a key tenet of our methodology is that the forecasts rapidly adjust to avoid systematic errors. The forecast evaluation exercise comparing our forecasts for U.S. states to others shows excellent performance for deaths, and average performance for cases.

One essential aspect of the forecasting methodology was to include seasonality in the forecasts. This came as a surprise to us as COVID‐19 is unlikely to act seasonally, although weekend effects could play a role. However, most countries have a weekly pattern in the reporting of cases and deaths, which matters greatly when forecasting, as was observed comparing our forecasts to those from LANL. Many models ignored the seasonal pattern of the data that can be costly in a forecasting context. The forecasts collected by the CDC, and its ensemble forecasts, always target a Saturday: ignoring seasonality could create biased forecasts, similar to what was shown for Brazil. We also question the accuracy of long‐term forecasts of COVID‐19 cases and deaths; many long‐term forecasts are much less reliable than their forecast intervals suggest.

In this article, we have emphasized the usefulness in forecasting of reported data on the pandemic when carefully treated to handle its many forms of nonstationarity. We know that forecasts will always be wrong to some extent: we are dealing with human behavior that is highly complex, partly unpredictable, facing unanticipated events, with limited information, and a lack of accurate data. But that does not mean that forecasts are not useful as we argue in our *Financial Times* “letter of the year”.^6^ Week‐ahead forecasts produced by us and other modeling groups help policymakers allocate health resources, inform mitigation policies, and manage expectations about the evolution of the pandemic. Hence, we believe that flexible and robust statistical methods for decomposing data and forecasting have a valuable role in the current pandemic.

## Footnotes

1 European Union refers to the 27 countries of the European Union with a population of 445 million people (this excludes the United Kingdom). The data for the United States include the 50 states, District of Columbia, and 5 territories, with a population of 330 million. LAC consists of the 26 borrowing countries of the Inter‐American Development Bank, although we omit Ecuador, leaving a population of about 620 million. The 25 countries included in LAC here are Argentina, Bahamas, Barbados, Belize, Bolivia, Brazil, Chile, Colombia, Costa Rica, Dominican Republic, El Salvador, Guatemala, Guyana, Haiti, Honduras, Jamaica, Mexico, Nicaragua, Panama, Paraguay, Peru, Suriname, Trinidad and Tobago, Uruguay, and Venezuela.

2 The United Kingdom consists of England, Scotland, Wales, and Northern Ireland. Each country adopted somewhat different mitigation policies. In terms of population, England is 84 percent of the United Kingdom.

3 Adjusted for population, in millions: Brazil 205, Mexico 128, Colombia 48, Argentina 43, Peru 33, and Chile 18.

4 We started publishing forecasts for Brazil on March 18, 2020, with a separate page for LAC from 25 April onward, with a frequency that is somewhat irregular at three to four updates a week. LANL produces two forecasts a week, with the last preforecast observation always on Sunday or Wednesday. LANL started providing forecasts for countries outside the United States on 26 April. Both are based on JH/CSSE data.

5 From JH/CSSE github csse_covid_19_daily_reports_us. We use the time‐series files of JH/CSSE, at csse_covid_19_time_series. These are revised later than the daily reports, so we have adjusted our forecast base to the daily reports.

6 See 〈www.ft.com/content/8684a104‐7a52‐11ea‐9840‐1b8019d9a987〉 (paywall).
